# A study on a fixed-bed for Pb(ii) removal by modified alkaline lignin-sodium alginate composite hydrogel

**DOI:** 10.1039/d4ra02975e

**Published:** 2024-07-26

**Authors:** Qiaorui Wang, Dingyun Liang, Yalan Yang, Yunran Zhang, Yirong Wang, Lilong Zhang, Rui Ma, Zhirui Niu

**Affiliations:** a Yan'an Key Laboratory of Agricultural Solidiste Resource Utilization, School of Petroleum Engineering and Environmental Engineering, Yan'an University 716000 P.R. China wang_qiaorui@foxmail.com niuzhirui@yau.edu.cn; b National Key Laboratory of Green Pesticide, Key Laboratory of Green Pesticide and Agricultural Bioengineering, Ministry of Education, State-Local Joint Laboratory for Comprehensive Utilization of Biomass, Guizhou University 550025 P.R. China; c Zhongsheng Environmental Technology Development Co., Ltd Xi'an 610000 P.R. China

## Abstract

In this work, alkaline lignin (AL) co-modified with trimercapto-*s*-triazine trisodium salt (TMT) and sodium alginate (SA) as a matrix were used to create a composite hydrogel for removing heavy metals, specifically divalent lead (Pb) from water. The obtained hydrogel beads were packed into a fixed bed, and then various operating conditions were explored to assess their impact on the efficiency of Pb(ii) removal. The findings indicated that the optimal removal efficiency for Pb(ii) was attained using an inflow rate of 0.159 L min^−1^, a hydrogel-II filling height of 40 cm, an initial Pb(ii) concentration of 10 mg L^−1^, and a bottom inflow direction. In the third adsorption–desorption cycle experiment, the breakthrough curve reached equilibrium after 650 min, in which equilibrium time for the initial breakthrough curve was 855 min, indicating that hydrogel-II exhibit good regeneration capability. This work serves as a foundation for practical applications in removing heavy metals from wastewater.

## Abstract 2

In this work, alkaline lignin (AL) co-modified with trimercapto-*s*-triazine trisodium salt (TMT) and sodium alginate (SA) as a matrix were used to create a composite hydrogel for removing heavy metals, specifically divalent lead (Pb) from water.

## Graphical abstract

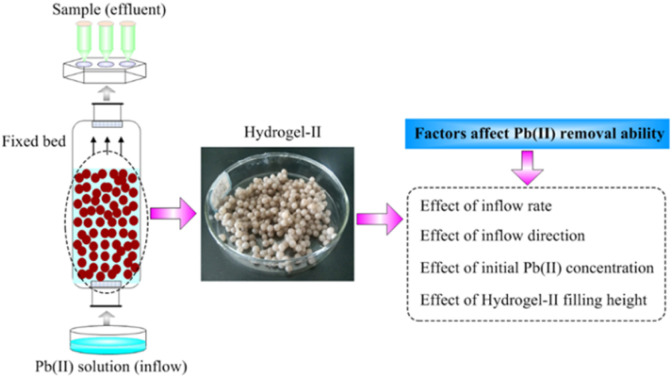

## 1 Introduction

Lead(ii) can be ingested into the human body *via* the food chain, leading to adverse effects on the nervous, hematopoietic (blood-forming), and digestive systems.^1^ Wastewater containing Pb(ii) mainly comes from industrial wastewater and acidic leachate from landfills, with discharge concentrations ranging from about 5 to 150 mg L^−1^.^2–5^ Presently, adsorption technology is extensively utilized for treating wastewater that contains Pb(ii). Compared to batch experiments of adsorption technology, fixed beds are more capable of concentrating on the treatment of wastewater containing Pb(ii) due to the continuous inflow model.^6,7^ In a fixed bed, when the Pb(ii) solution is pumped through the bed, it is adsorbed onto its adsorbent. The performance of the adsorbent in the fixed bed is typically studied using the breakthrough curves, including influent concentration, inflow rate, fixed bed height, and the direction of water inflow.^8^ Furthermore, the breakthrough time in a fixed bed is also an important parameter determining its operation and dynamic response. This clearly demonstrates that a well-thought-out design of the fixed bed can offer fundamental data for industrial application.^9^

In batch adsorption experiments, current reports mainly involve the usage of different types of modified lignin to remove Pb(ii) from water.^10^ However, this modified lignin is typically in the form of fine or ultrafine powder, making it impractical for use in fixed beds or other flow-through systems with high pressure drops. To overcome this problem, an adsorbent derived from modified lignin powder can be encapsulated in a porous carrier, such as sodium alginate (SA) hydrogel, to reduce the loss of powder adsorbent during the operation of fixed beds.^11,12^ SA, it a natural polysaccharide derived from brown algae, has been engineered as a drug encapsulation matrix because of its safety and excellent film-forming abilities.^13^ In addition to the above properties, SA can also undergo ion exchange with divalent cations (such as Ca^2+^) to form hydrogels. This material, with its three-dimensional structure, can enhance permeability and has been widely applied in recent years for adsorption and pollutants removal.^14^ Currently, the main research focuses on preparing SA as hydrogels for batch adsorption experiments to remove anions (phosphate or fluoride),^15,16^ heavy metal oxyanions (arsenate or arsenite),^17^ and dyes (Acid Black 172 or methylene blue).^18^ However, no studies have reported using SA-encapsulated modified lignin in fixed beds for heavy metal removal.

The TMT known for its strong chelating ability with heavy metals like Pb(ii), Cu(ii), Cd(ii), Ni(ii), and Hg(ii) in aqueous solutions,^19^ was chosen to modify AL to enhance removal capability. This study further encapsulated TMT-modified alkaline lignin (AL) within an SA matrix to create a composite hydrogel (hydrogel-II), and then filled it into a fixed bed. The study studied influence of factors include inflow rate, inflow direction, initial Pb(ii) concentration, and hydrogel-II filling height on Pb(ii) removal. The Dose–response, Thomas, and Yoon–Nelson mathematical models were established to characterize the kinetic behavior. Moreover, the practical applications of hydrogel-II can be assessed through three consecutive adsorption–desorption cycle experiments. This research provides a theoretical basis for evaluating use potential of hydrogel-II in the real-world to remove heavy metals from water or wastewater.

## 2 Materials and methods

### 2.1 Experimental instruments and reagents

The experiment utilized pH meter (FE20, Mettler-Toledo Instruments Co., Ltd), organic glass column (Guangzhou Yuanteng Environmental Technology Co., Ltd), ultrapure water equipment (CD-UPW-II, Chengdu Yuechun), peristaltic pump (BT300X, Changzhou Kejian Peristaltic Pump), temperature-controlled magnetic stirrer (85-2, Changzhou Guohua Electric Appliance Co., Ltd), scanning electron microscope (SEM, Verios 5 XHR, Thermo Fisher Scientific, USA), and inductively coupled plasma optical emission spectrometer (ICP-OES, ICPS-751, Shimadzu, Japan).

Alkaline lignin (AL, black powder), trisodium trimercaptotriazine (TMT), potassium iodide, and elemental iodine, all analytical grade (Shanghai Jizhi Biochemical Technology Co., Ltd, China). Hydrochloric acid (37 wt%) and sodium hydroxide of analytical grade were obtained from Tianjin Fuyu Fine Chemical Co., Ltd. Sodium alginate (SA) and anhydrous calcium chloride, also of analytical grade, were sourced from Tianjin Fuchen Chemical Reagents Factory. The lead nitrate standard solution (1000 mg L^−1^) was used as is, without any further treatment (National Standards Testing and Certification Co., Ltd). Ultrapure water (resistivity = 18 × 10^6^ Ω cm^−1^) was used throughout the test.

### 2.2 Adsorbent preparation

#### 2.2.1 FAL preparation

The 1.0 g of AL was placed into a 500 mL beaker, accompanying by 15 mL of TMT solution and 50 mL of ultrapure water were then added. The mixture was stirred at room temperature for 120 min using a magnetic stirrer. Then the beaker was placed into a 0 °C refrigerator for 3 h, stirring manually before slowly adding 15 mL of saturated KI-I_2_ solution drop by drop through a rubber bulb pipette, and letting the mixed solution stand. After 3 h, the beaker was removed from the refrigerator and continued to be stirred on a magnetic stirrer at room temperature for 12 h. The obtained mixture was vacuum filtered and rinsed repeatedly with ultrapure water until the filtrate was colorless. Finally, the solid substance was collected and dried under vacuum freeze-drying at −60 °C for 5 h, yielding a gray powder adsorbent (FAL).

#### 2.2.2 Hydrogel-II preparation

The 5.0 g of SA was placed it into a 500 mL beaker, then added 250 mL of ultrapure water. Set the beaker on a magnetic stirrer and stirred at 60 °C for 5 h to dissolve the SA. The 0.4 g of FAL was added it to the beaker, stirring for another 5 h to ensure thorough mixing with the SA solution. The 16.0 g of CaCl_2_ was added into a 2000 mL beaker with 500 mL of ultrapure water to prepare a CaCl_2_ solution. Gradually dropped the FAL and SA mixture into CaCl_2_ solution using a rubber bulb pipette to form spherical beads. Allowed the beads and CaCl_2_ solution to sit for 24 h to ensure complete cross-linking of the beads. Finally, these beads were rinsed with ultrapure water until pH value of the final rinse solution reached 6.5. The obtained beads were used as composite hydrogel adsorbent, which was referred to as hydrogel-II. This adsorbent had a particle size ranging from 0.3 to 0.5 cm.

### 2.3 Fixed bed construction

**Fig. 1:**
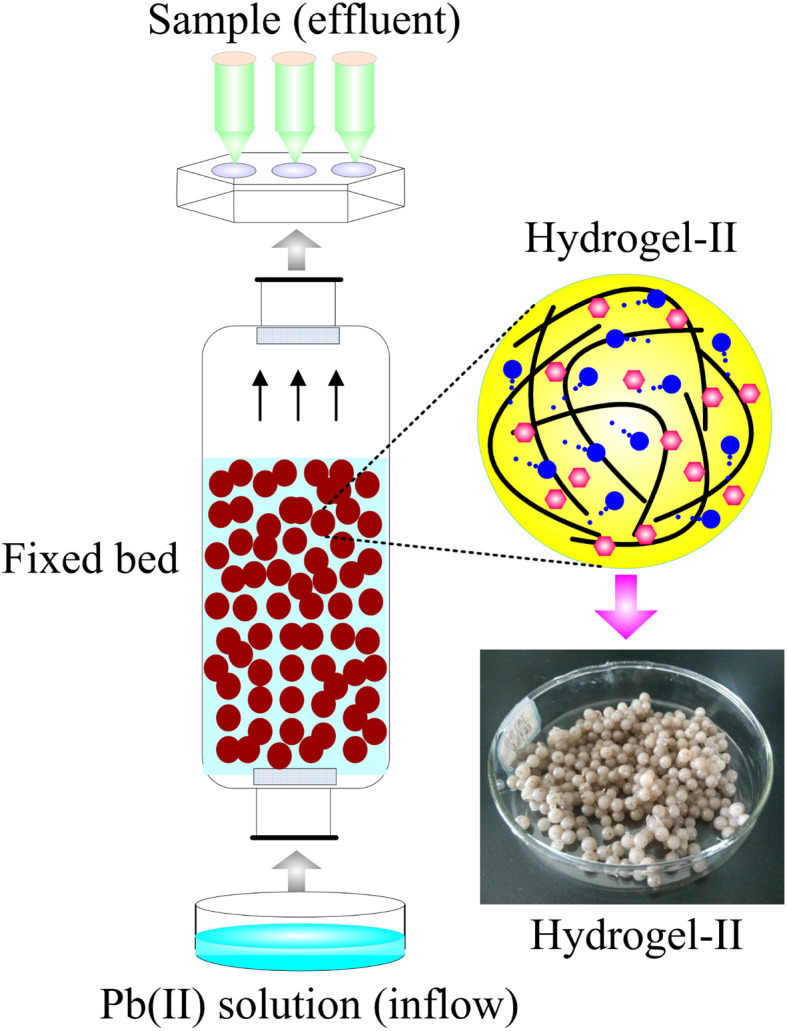
The schematic diagram of fixed bed.

The fixed bed was constructed through an organic glass column (length = 65 cm, diameter = 5 cm). To prevent the loss of beads with the water inflow during operation, nuts with sieves were installed at both ends of the column. The inflow silicone tubing applied in the fixed bed was connected to the column with fittings. The beads were loaded into the glass column before establishing the connection. The Pb(ii) solution inflow was pumped from a supply tank located at the column's base using a peristaltic pump. The fixed bed operated at room temperature, with pH value of the Pb(ii) solution adjusted to 6.0 using HCl or NaOH (0.1 M). Fig. 1 presents a schematic representation of the fixed bed.

### 2.4 Fixed bed operation experiments

During the operation of the fixed bed, the studied parameters included inflow rate (*Q*, L min^−1^), inflow direction (bottom inflow and top inflow), initial Pb(ii) concentration (*C*_0_, mg L^−1^), and hydrogel-II filling height (*Z*, cm). Breakthrough curves were generated by plotting the ratio of outlet to inlet Pb(ii) concentrations (*C*_*t*_/*C*_0_, where *C*_0_ represents the initial Pb(ii) concentration and *C*_*t*_ is the concentration at time *t*) against reaction time (*t*, min).

#### 2.4.1 Effect of inflow rate

The Pb(ii) solution inflow rates were set at 0.159, 0.255, and 0.318 L min^−1^, with other parameters fixed (*C*_0_ = 10 mg L^−1^, hydrogel-II filling height = 40 cm, solution pH = 6.0, and bottom inflow). Based on the study of Pb(ii) removal at solution pH values of 1.0, 3.0, 4.0, 5.0, and 6.0 in the batch experiment, accompanying by considering Pb(ii) species at different pH levels, it was noted that adjusting solution pH to 6.0 enhanced Pb(ii) removal efficiency while preventing Pb(OH)_2_ precipitation.^20,21^

#### 2.4.2 Effect of inflow direction

The inflow direction was varied between top and bottom, while keeping other parameters constant (solution pH = 6.0, hydrogel-II filling height = 40 cm, *C*_0_ = 10 mg L^−1^, and inflow rate = 0.159 L min^−1^).

#### 2.4.3 Effect of initial Pb(ii) concentration

Initial Pb(ii) concentrations were 5, 10, and 20 mg L^−1^, with other parameters fixed (solution pH = 6.0, hydrogel-II filling height = 40 cm, bottom inflow, and inflow rate = 0.159 L min^−1^).

#### 2.4.4 Effect of hydrogel-II filling height

Filling heights were 20 (*m* = 10 g), 40 (*m* = 20 g), and 60 cm (*m* = 40 g), with other parameters held constant (solution pH = 6.0, bottom inflow, *C*_0_ = 10 mg L^−1^, and inflow rate = 0.159 L min^−1^). The total sampling time was 1040 min, with samples taken every 8 min from 0 to 80 min, every 40 min from 80 to 720 min, and every 80 min from 720 to 1040 min. These samples were collected and analyzed for Pb(ii) concentration using ICP-OES.

### 2.5 Analysis of fixed bed operation

#### 2.5.1 Fixed bed data analysis

The adsorption capacity of the fixed bed at breakthrough time (*t*_b_) or saturation time (*t*_s_), denoted as *q*_c_ (mg), is usually the capacity at *t*_s_.^22^ The *t*_b_ in the fixed bed is the time required for the effluent Pb(ii) concentration to reach 1.0 mg L^−1^. The operation time is defined as *t*_s_ when *C*_*t*_/*C*_0_ = 0.90, indicating the complete loss of hydrogel-II's treatment capacity.1
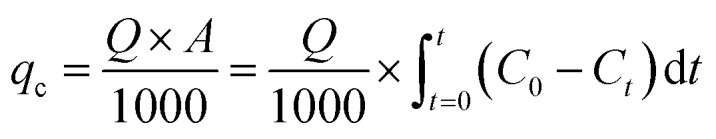
where *Q* represents the inflow rate (mL min^−1^); *A* is the area under the breakthrough curve; *t* stands for breakthrough time (*t*_b_) or saturation time (*t*_s_) (min); *C*_0_ is Pb(ii) initial concentration (mg L^−1^); *C*_*t*_ is Pb(ii) concentration in effluent at time *t* (mg L^−1^).

*q* (mg g^−1^) represents Pb(ii) amount uptake by per unit mass of adsorbent in the fixed bed:^22^2
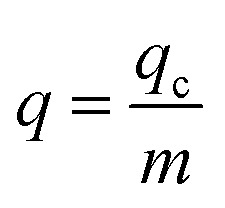
where *m* refers to hydrogel-II mass loaded in the fixed bed (g).

*M* (mg) represents the total amount of Pb(ii) solution introduced into the fixed bed:^22^

3 *M* = *C*_0_ × *Q* × *t*_s_

*V*_eff_ is the effluent Pb(ii) volume from the fixed bed at time *t*_s_.^22^*Y* denotes the overall removal efficiency of Pb(ii):^22^

4 *V*_eff_ = *Q* × *t*

5
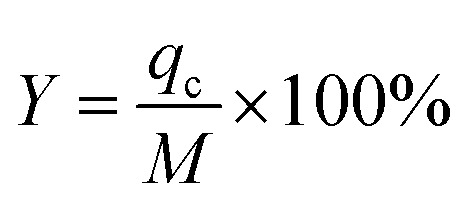


To study the impact of bed height, a Bed Depth Service Time (BDST) model formulated from the simplified Bohart-Adams model was used. The BDST model was applied to investigate the impact of different filling heights on Pb(ii) removal:^23^6
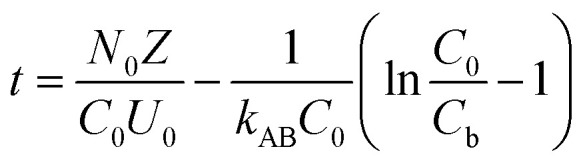
where *N*_0_ belongs to adsorption capacity of the fixed bed (mg L^−1^); *Z* represents the filling height of the fixed bed (cm); *U*_0_ is the superficial velocity calculated as inflow rate divided by the cross-sectional area of the column (cm min^−1^); *k*_AB_ is rate constant (L mg^−1^ min^−1^); *C*_b_ is Pb(ii) concentration in effluent at breakthrough point (mg L^−1^).

When defining the column service time as the duration required for the effluent Pb(ii) concentration to attain 1.0 mg L^−1^, the minimum theoretical depth of the fixed bed necessary to keep the effluent concentration below the breakthrough level at *t* = 0 min is calculated. This is known as the critical height (*Z*_0_) of the fixed bed:^23^7
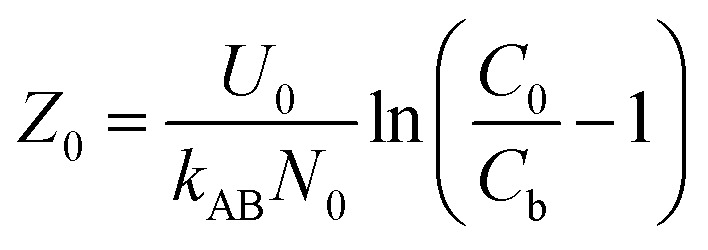


Moreover, the BDST model curve was transformed into a linear equation, *y* = *ax* + *b*, where *y* represents the service time (min), *x* is the filling height of the fixed bed in centimeters, *a* is the slope, and *b* is the intercept. Here, the slope (*a*) = *N*_0_/*C*_0_ × *U*_0_, and the intercept (*b*) = −1/*k*_AB_ × *C*_0_[ln(*C*_0_/*C*_b_ − 1)].^24^ Therefore, *N*_0_ and *k*_AB_ can be calculated from the slope and intercept of the line, respectively.^24^

#### 2.5.2 Fixed bed breakthrough curve model

As an empirical model, the Dose–response model is widely applied to describe the kinetic properties and behavior of the fixed bed columns, especially in adsorption experiments targeting heavy metals:^25^8
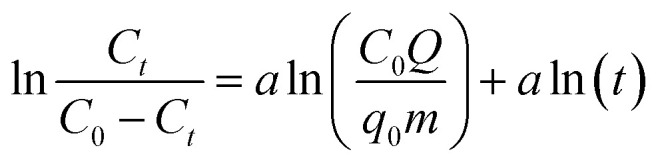
where *q*_0_ represents the maximum adsorption capacity (mg g^−1^), and *a* is the model constant.

The Thomas model is among the most extensively utilized and frequently applied theories in understanding the performance of fixed bed columns.^24^ This model is based on Langmuir kinetics and disregards axial dispersion.^24^9
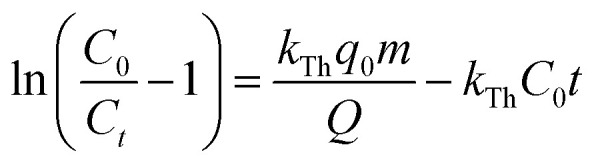
where *k*_Th_ represents the model constant (mL min^−1^ mg^−1^).

The Yoon–Nelson model posits that the rate at which the adsorption probability for each adsorbate molecule decreases is directly proportional to both the probability of adsorbate adsorption and the occurrence of adsorbate breakthrough on the adsorbent.^26^10
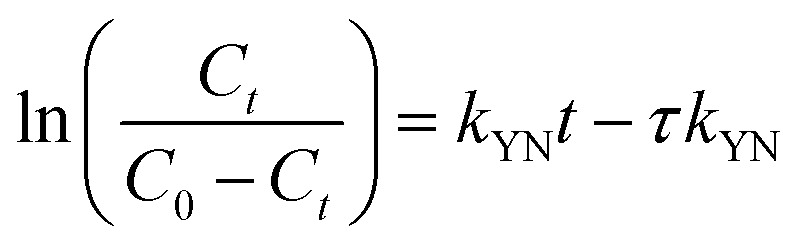
where *τ* represents the time (min) required for *C*_*t*_/*C*_0_ to reach 50% breakthrough; *k*_YN_ is the model constant (L min^−1^).

#### 2.5.3 Adsorption–desorption cycling experiment

In the cycling experiment, the fixed bed operation conditions for the adsorption experiments were as follows: initial Pb(ii) concentration (*C*_0_) of 10 mg L^−1^, solution pH of 6.0, reaction temperature of 25 °C, bottom inflow, hydrogel-II filling height of 40 cm (20 g), and inflow rate of 0.159 L min^−1^. Sampling was conducted over a period of 1040 min, with samples collected every 8 min from 0 to 80 min, every 40 min from 80 to 720 min, and every 80 min from 720 to 1040 min. Samples were collected and analyzed for Pb(ii) concentration using ICP-OES.

During desorption cycle experiment, HCl was used as the regenerating agent to remove Pb(ii) from hydrogel-II surface. Other operating conditions were consistent with the adsorption experiment. Sampling was carried out over 240 min, with samples collected every 8 min and analyzed for Pb(ii) concentration using ICP-OES. After each adsorption cycle, hydrogel-II was desorbed with HCl and washed with tap water until the influent solution reached neutral pH. The cleaned hydrogel-II was then applied for the next adsorption experiment. The regeneration experiment was conducted for three cycles. In the cycling experiment, adsorption data were plotted using *C*_*t*_/*C*_0_ to generate breakthrough curves. Desorption capacity was evaluated using individual adsorption amount, total adsorption amount, individual desorption efficiency, and cumulative desorption efficiency.

11 *q*_1_ = (*C*_0_ − *C*_*t*_) × *V*_1_

12
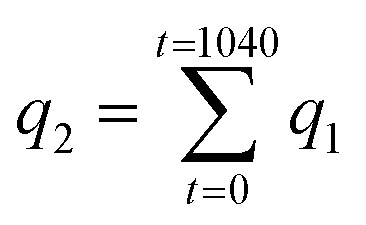

13 *q*_3_ = *C* × *V*_1_

14
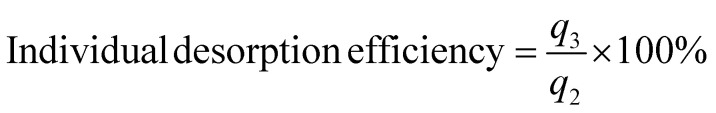
15
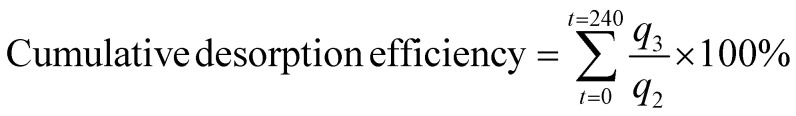

16 *V*_1_ = π*r*^2^*h*

where *q*_1_ represents the individual adsorption amount (mg); *q*_2_ is the total adsorption amount (mg); *q*_3_ is the individual desorption amount (mg); *C* is Pb(ii) concentration in the effluent during desorption process (mg L^−1^); *V*_1_ denotes the volume of fixed bed (L).

### 2.6 Scanning electron microscopy (SEM) measurement

Before measuring hydrogel-II, it is placed on a conductive silicone pad, and gold powder is sprayed onto its surface using an ion sputtering coater. During the measurement process, the electron beam acceleration voltage is set to 20.00 kV, and scanning electron microscope images of the collected samples are obtained.

### 2.7 Fourier transform infrared (FT-IR) spectroscopy

The sample hydrogel-II/-Pb(ii) and potassium bromide (KBr) were placed in a forced air drying oven at a temperature of 80 °C for 3 h. Afterward, the sample and KBr were allowed to cool to 40 °C, removed, and placed in a vacuum bag, then cooled to room temperature (25 °C). Subsequently, a small amount of the sample was taken and placed in a mortar with KBr, ground to a powder, pressed into pellets, and the FT-IR spectrum was measured. The measurement conditions were as follows: resolution of 4 cm^−1^, covering a wavelength range from 500 to 4000 cm^−1^. In adsorption–desorption cycling experiment for FT-IR measurement, samples were taken for adsorption at 8, 720, and 1040 min, and for desorption at 8, 160, and 200 min. Digital photographs of the obtained samples were taken using a digital camera to observe the morphological changes of hydrogel-II beads during three consecutive regeneration cycles.

## 3 Results and discussion

### 3.1 Hydrogel-II preparation

**Fig. 2:**
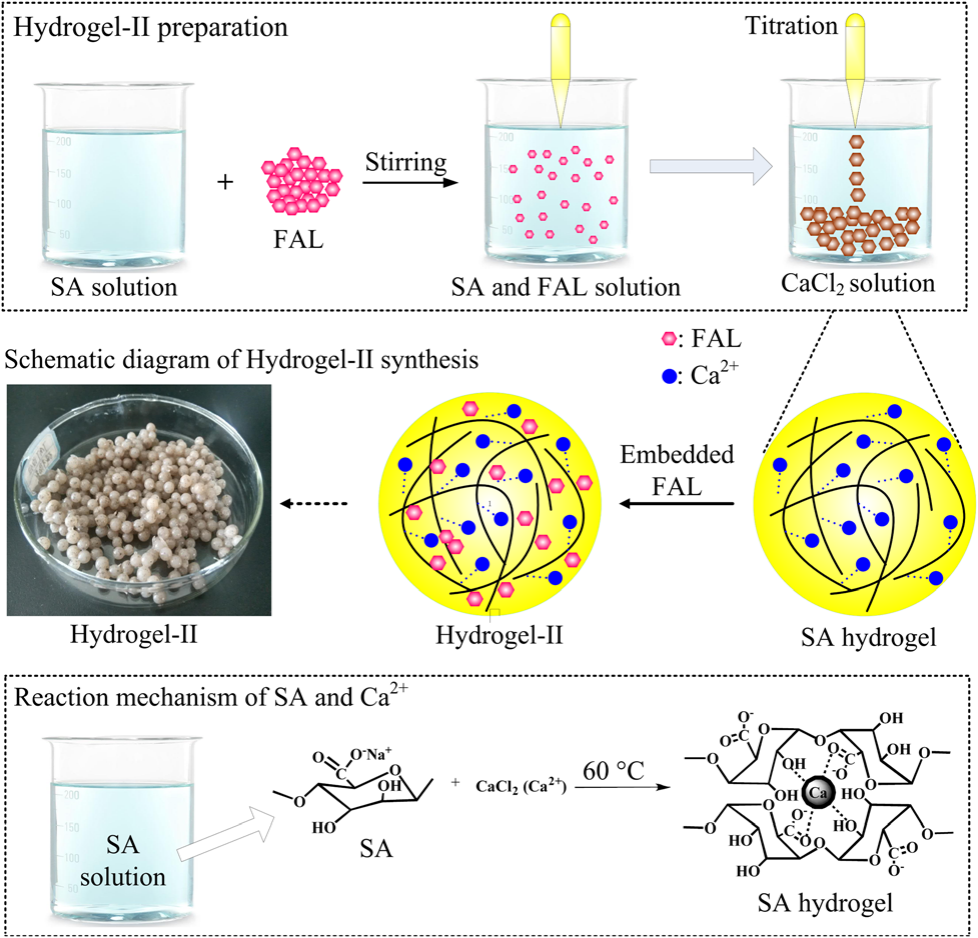
Synthetic scheme of hydrogel-II and reaction mechanism.

**Fig. 3:**
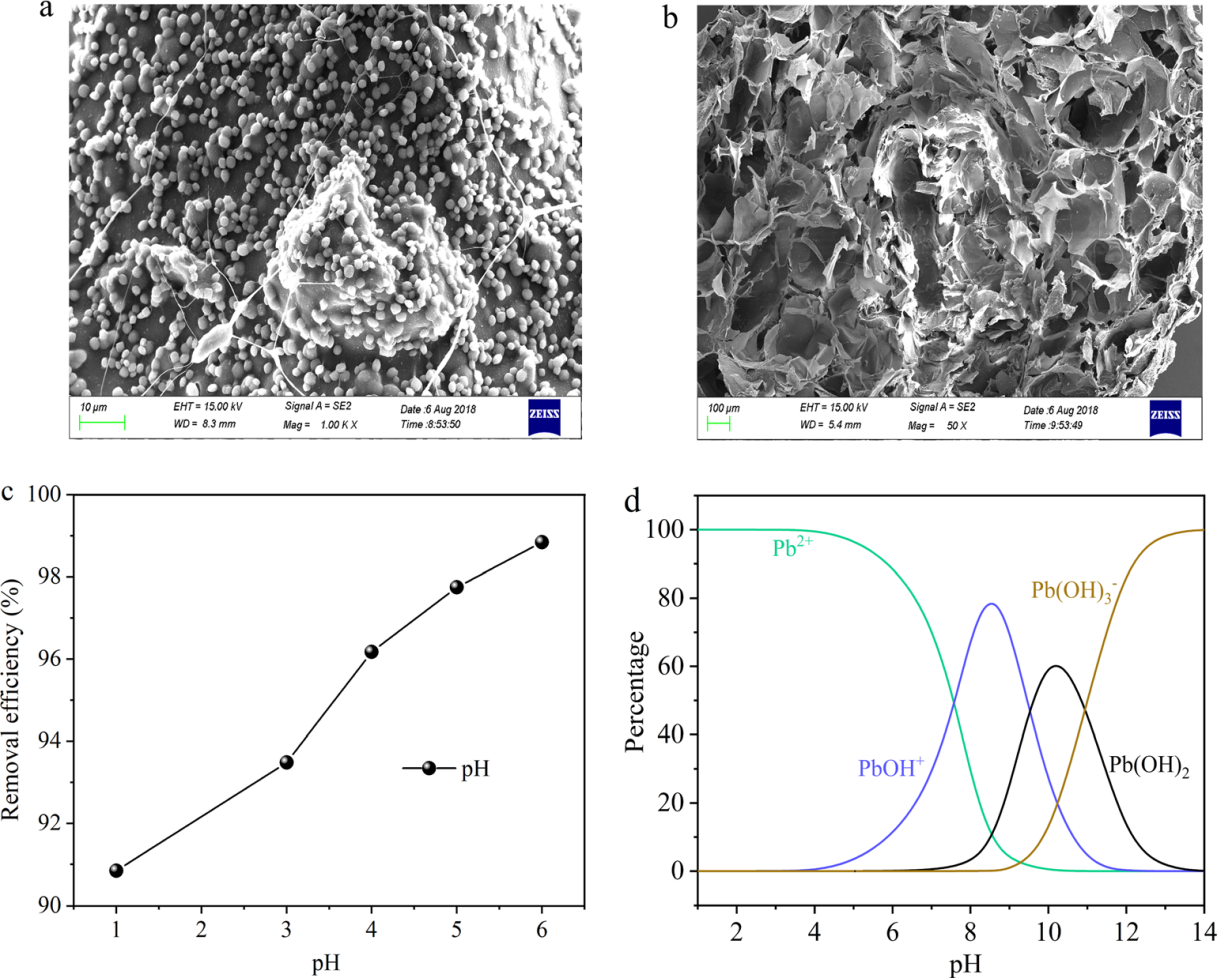
(a) SEM image of hydrogel-II surface; (b) hydrogel-II planning surface; (c) solution pH impacted on Pb(ii) removal efficiency; and (d) Pb(ii) species at different pH values.

SA is a natural polysaccharide, comprising molecules linked by (1–4) β-d-mannuronic acid (M) and α-l-guluronic acid (G) connections. Due to the presence of a large amount of carboxyl groups (–COOH), SA exhibits polyanionic behavior in the aqueous solutions. Moreover, SA can undergo ion exchange reactions with divalent metal cations (such as Ca^2+^, Fe^2+^, and Sr^2+^) through Na^+^ in the G units.^27^ Due to the sugar rings of the G units being in the ^1^C_4_ conformation, the polymer chains can form a zigzag pattern with Ca^2+^ ions, creating sack-like cavities,^27^ which can be described as the egg-box model (Fig. 2). Based on the reaction mechanism described above, SA can undergo an ion exchange reaction with CaCl_2_ solution, thereby preparing SA hydrogel. So this paper embedded FAL powder in SA to prepare SA-based hydrogel beads, named hydrogel-II (Fig. 2). Furthermore, it shows that hydrogel-II surface contains some fine beads (Fig. 3a), while displays its cross-section as a porous structure (Fig. 3b), is consistent with the structure of sack-like cavities.

In prior batch adsorption studies, the primary focus was on investigating the removal capacity of hydrogel-II for Pb(ii).^28^ The findings suggested that the pseudo-second-order kinetics and Langmuir isotherm model provided a better fit for the experimental data, indicating that the adsorption of Pb(ii) onto hydrogel-II aligns with chemical adsorption and monolayer adsorption mechanisms.^28^ In the investigation of Pb(ii) removal by hydrogel-II, the influence of solution pH on its efficiency was also studied (Fig. 3c). The point of zero charge for hydrogel-II was determined to be 3.5.^28^ So it was observed that as the solution pH lower than 3.5 (1.0 and 3.0), respectively, accompanied with lower removal efficiencies (90.8 and 93.5%). At lower pH, the removal efficiency was low because the protons (H^+^) can compete with the limited active sites of hydrogel-II for Pb(ii), thereby reducing the removal efficiency.^29^ However, when the solution pH increased (4.0 to 6.0), the competition of H^+^ with Pb(ii) for active sites decreased, in which the groups in hydrogel-II undergo deprotonation, thereby enhancing the removal efficiency (96.2 to 98.8%).^29^ Additionally, the Pb(ii) precipitation would occur when solution pH exceeds 7.2.^30,31^ Combining the different distribution species of Pb(ii) obtained using Visual MINEQL 3.0 software at various pH values (Fig. 3d), Pb(ii) primarily exists as Pb^2+^ ions in the aqueous solution when the pH ranges from 1.0 to 6.0, with a small amount present as Pb(OH)^+^. As described as above, when solution pH exceeds 7.0, the Pb(OH)_2_ precipitation occurs. Therefore, to prevent Pb(OH)_2_ precipitation from affecting the experimental results, the pH of the Pb(ii) influent solution was adjusted to 6.0. This paper further explores the removal capacity of hydrogel-II for Pb(ii) by filling it in a fixed bed.

### 3.2 Fixed bed operation experiment

#### 3.2.1 Effect of inflow rate

**Fig. 4:**
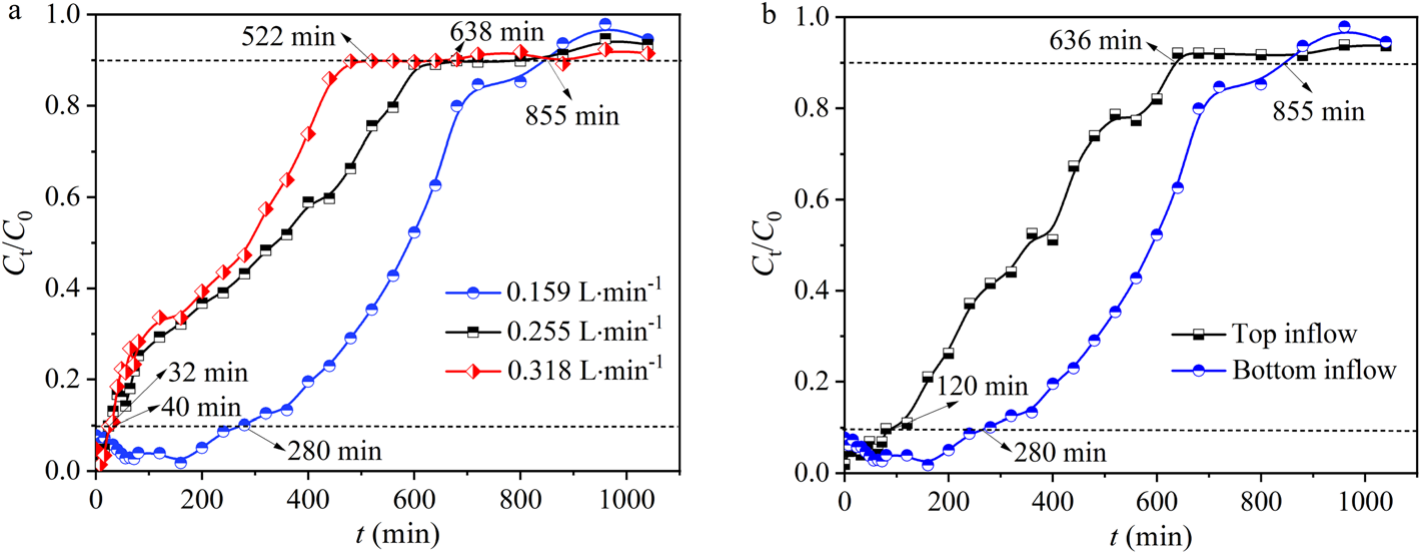
(a) The impact of inflow rates and (b) inflow direction.

The inflow rate is a critical parameter as it regulates the contact time between the solute and adsorbent surface. In this study, the inflow rates were set at 0.159, 0.255, and 0.318 L min^−1^. It was discovered that as the inflow rate increased, the residual concentration of Pb(ii) in the effluent from the fixed bed showed a gradual increasing trend (Fig. 4a). At a lower inflow rate of 0.159 L min^−1^, the curve more closely resembles an S-shaped breakthrough curve, indicating a slower adsorption process. At the same time, when the inflow rate increased from 0.159 to 0.318 L min^−1^, the breakthrough time (*t*_b_) of the fixed bed decreased from 280 to 32 min, as well as the saturation time (*t*_s_) decreased from 855 to 522 min (Fig. 4a and Table 1). Concurrently, as the inflow rate increased, the adsorption capacity (*q*) at *t*_s_ decreased from 42.2 to 39.4 mg g^−1^, accompanied by the removal rate (*Y*) decreasing from 62.1% to 47.4% (Table 1). Moreover, when the inflow rate increased from 0.159 to 0.318 L min^−1^, the total amount of Pb(ii) entering the fixed bed (*M*) increased from 1359 to 1659 mg, and the effluent volume (*V*_eff_) at *t*_s_ also increased from 135 to 165 L. The results indicated that as the inflow rate increases, even though both the total amount of Pb(ii) entering the fixed bed (*M*) and the effluent volume (*V*_eff_) increase, the accelerated movement of Pb(ii) through the fixed bed results in insufficient residence time of Pb(ii) in the column, thereby leading to a decrease in Pb(ii) adsorption capacity and removal efficiency, as well as a decrease in breakthrough and penetration times.^32^

**Table 1:** Operating parameters for fixed bed inflow rate and inflow direction

Parameters	Inflow rates (L min^−1^)			Inflow direction	
	0.159	0.255	0.318	Top inflow	Bottom inflow
*t*_b_ (min)	280	40	32	120	280
*t*_s_ (min)	855	638	522	636	855
*q*_c_ (mg)	844	795	788	314	844
*M* (mg)	1359	1626	1659	1011	1359
*q* (mg g^−1^)	42.2	39.7	39.4	15.7	42.2
*V*_eff_ (L)	135	162	165	101	135
*Y* (%)	62.1	48.8	47.4	31.1	62.1

#### 3.2.2 Effect of inflow direction

The inflow direction (bottom inflow and top inflow) in a fixed bed significantly affects the performance of hydrogel-II for Pb(ii) removal.^33^ With bottom inflow, the upward movement of water can enhance contact time between Pb(ii) and hydrogel-II, leading to more efficient adsorption.^33^ This direction helps in preventing channeling and ensures uniform distribution of contaminants, utilizing the active sites of hydrogel-II more effectively.^33^ In addition, bottom inflow also helps in better management of pressure drop and maintains consistent inflow rates, enhancing the mass transfer rates and ensuring thorough Pb(ii) uptake by hydrogel-II. Conversely, top inflow can lead to quicker saturation at the upper layers, reducing overall efficiency and creating dead zones. In this work, by controlling the direction of the Pb(ii) solution inflow, the solution was pumped into the fixed bed from the bottom (bottom inflow) and the top (top inflow) using a peristaltic pump. When the inflow direction was from the top, the breakthrough time (*t*_b_) of the fixed bed was 120 min, and the saturation time (*t*_s_) was 636 min (Fig. 4b and Table 1). When the inflow direction was from the bottom, the *t*_b_ was determined to be 280 min, and *t*_s_ was 855 min (Fig. 4b and Table 1), finding that the times were longer compared to top inflow, indicating a more thorough contact between the Pb(ii) solution and hydrogel-II in the fixed bed. Also, when the inflow direction was from the bottom, the adsorption capacity (*q*) of the fixed bed at *t*_s_ was 42.2 mg g^−1^, higher than the 15.7 mg g^−1^ when inflow direction was from the top. Furthermore, when inflow direction was from the bottom, the removal efficiency (*Y*) of fixed bed was 62.1%, the total amount of Pb(ii) entering the fixed bed (*M*) (1359 mg), and the effluent volume (*V*_eff_) at *t*_s_ (135 L) were also higher (Table 1), compared to the top inflow where *Y*, *M*, and *V*_eff_ were 31.1%, 1011 mg, and 101 L, respectively. The results indicate that when the Pb(ii) inflow direction is from the bottom, it can increase the contact time between the Pb(ii) solution and hydrogel-II in the fixed bed, which is beneficial for extending the breakthrough and penetration times, thereby improving the removal capacity for Pb(ii).

#### 3.2.3 Effect of initial Pb(ii) concentration

**Fig. 5:**
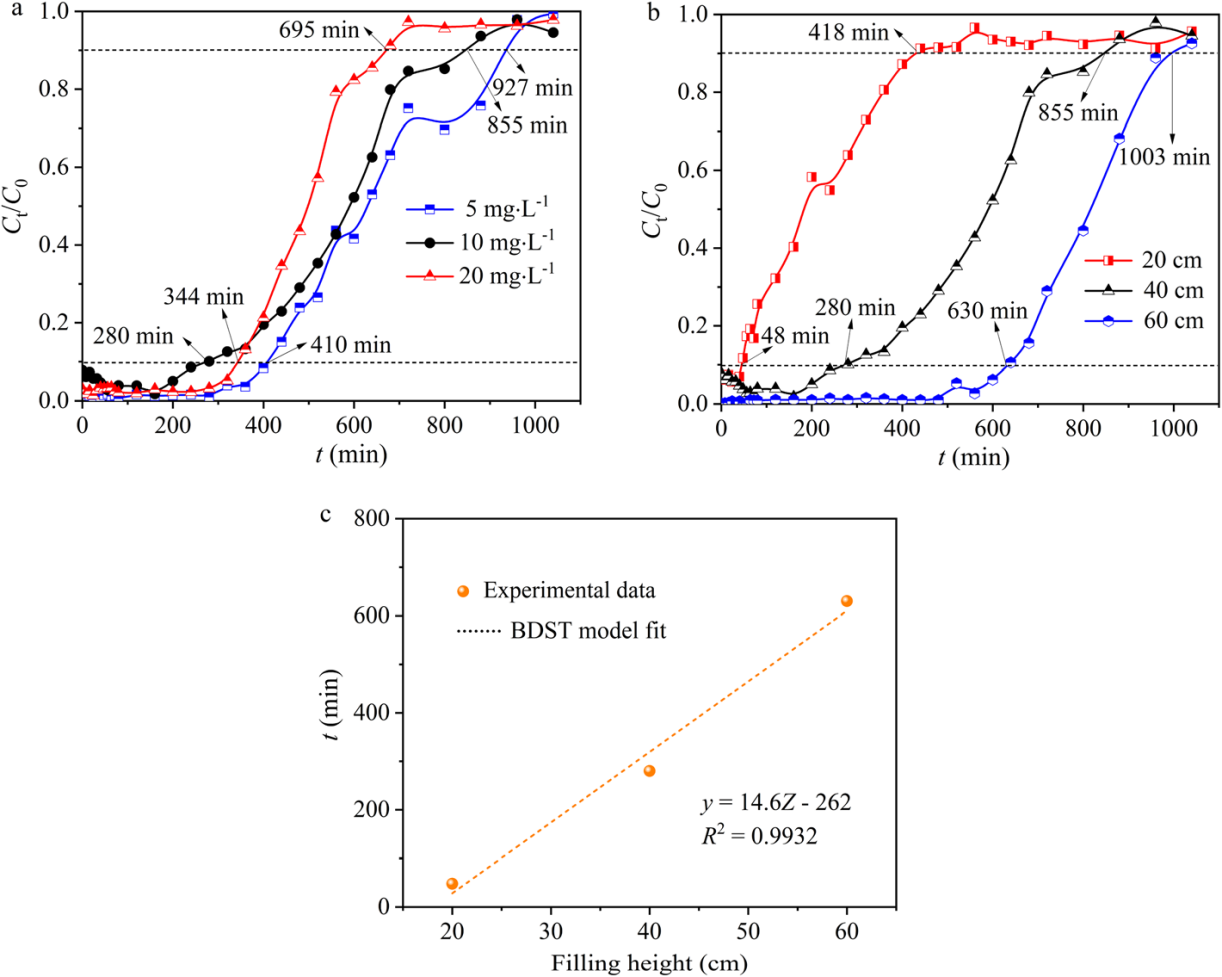
(a) The effect of initial Pb(ii) concentration and (b) hydrogel-II filling height. (c) The BDST model.

The impact of Pb(ii) concentration on adsorption capacity is also a crucial factor, indicating that a given mass of adsorbent can remove only a specific quantity of Pb(ii). As the initial Pb(ii) concentration rose from 5 to 20 mg L^−1^, there was a slight increase in the slope of the breakthrough curve from the breakthrough point to the saturation point (mass transfer zone), accompanied by a decreasing trend in both *t*_b_ (breakthrough time) and *t*_s_ (saturation time) (Fig. 5a and Table 2). When the initial concentration of Pb(ii) was 20 mg L^−1^ and 10 mg L^−1^, *t*_b_ was 344 and 280 min, respectively, and *t*_s_ was 695 and 855 min, respectively, both lower than the values at 5 mg L^−1^, which were *t*_b_ (410 min) and *t*_s_ (927 min) (Fig. 5a and Table 2). It is commonly assumed that higher initial concentrations would lead to shorter breakthrough times; however, it is essential to recognize that the observed outcome may be influenced by a variety of factors, including variations in mass transfer kinetics, saturation effects, and the adsorption capacity of hydrogel-II at different initial concentrations. When Pb(ii) initial concentrations were 5, 10, and 20 mg L^−1^, the saturated Pb(ii) adsorption amounts at *t*_s_ corresponded to 24.5, 42.2, and 60.5 mg g^−1^. With an inflow Pb(ii) concentration at 5 mg L^−1^, the removal rate (*Y*) of the fixed bed for Pb(ii) was 66.7%, higher than at 10 mg L^−1^ (62.1%) and 20 mg L^−1^ (54.7%). When Pb(ii) initial concentrations were 5, 10, and 20 mg L^−1^ (Table 2), the effluent volumes (*V*_eff_) at *t*_s_ were 147, 135, and 110 L, respectively, showing a gradually decreasing trend in treatment volume as the Pb(ii) concentration increased. The findings suggested that the higher initial concentrations of Pb(ii), the curve is steeper, and the solution treated is less.^34^ Nevertheless, at lower initial Pb(ii) concentrations, the breakthrough curve extends further, indicating a greater volume of solution treated.^35^ Therefore, at higher initial concentrations of Pb(ii), the concentration gradient increases, creating a more potent mass transfer driving force.^36^ This resulted in a faster migration speed of the solute within the column and a quicker saturation of the adsorption sites on hydrogel-II surface.

**Table 2:** Operating parameters for Pb(ii) initial concentration and filling height

Parameters	Initial Pb(ii) concentration (mg L^−1^)			Filling height (cm)		
	5	10	20	20	40	60
*t*_b_ (min)	410	280	344	48	280	630
*t*_s_ (min)	927	855	695	418	855	1003
*q*_c_ (mg)	491	844	1210	350	844	1277
*M* (mg)	736	1359	2210	664	1359	1594
*q* (mg g^−1^)	24.5	42.2	60.5	35.0	42.2	42.6
*V*_eff_ (L)	147	135	110	66	135	159
*Y* (%)	66.7	62.1	54.7	52.7	62.1	80.1

#### 3.2.4 Effect of hydrogel-II filling height

The packing height of hydrogel-II in the fixed bed was 20 (10), 40 (20), and 60 cm (40 g). When increasing the packing height of hydrogel-II in the fixed bed, the breakthrough curve shifts to the right and becomes smoother, while the slope of the curve decreases. When the packing height decreased from 60 to 20 cm, the value of *t*_b_ decreased from 630 to 48 min, accompanied by *t*_s_ decreasing from 1003 to 418 min (Fig. 5b and Table 2). This is because a higher packing height means a greater mass of adsorbent, leading to more available active sites for adsorption.^35,37^ The extension in breakthrough time results from the increased distance and duration of mass transfer zone movement at both ends of the fixed bed, attributed to a higher packing layer.^35,37^ When the packing heights of the fixed bed were 20, 40, and 60 cm, the saturated adsorption capacities (*q*) were 35.0, 42.2, and 42.6 mg g^−1^, respectively (Table 2). Furthermore, when the packing height increased from 20 to 60 cm (Table 2), the Pb(ii) effluent volume (*V*_eff_) at *t*_s_ increased from 66 to 159 L, and the value of *Y* for Pb(ii) also increased from 52.7 to 80.1%. The results indicated that the smaller the packing height of the fixed bed layer, the earlier it exhausts. However, the adsorption amounts of Pb(ii) for different fixed bed packing heights are similar, suggesting that increasing the packing depth of the fixed bed does not have a significant impact on the dynamic adsorption of Pb(ii). The increase in packing height can also be interpreted as an increase in the mass of hydrogel-II, which provided more active sites for the removal of Pb(ii).

The BDST model was utilized to delve deeper into the adsorption capabilities of hydrogel-II in the fixed bed column for Pb(ii). In this model, the correlation coefficient (*R*^2^) is 0.9932 (Fig. 5c), indicating that the model fits the experimental data well. The calculated adsorption capacity (*N*_0_) is determined to be 1182 mg L^−1^, and the *k*_AB_ value is 0.003 L mg^−1^ min^−1^. A higher *N*_0_ value indicates faster mass transfer speed and higher adsorption efficiency of the fixed bed for Pb(ii).^22^ Hence, a larger *k*_AB_ value indicates that the influence of packing height on the adsorption capacity for Pb(ii) is less significant.^22^ Based on a 10% breakthrough, the minimum bed depth (*Z*_0_) was calculated to be 11.6 cm, which is lower than the packing height of 20 cm, indicating a high Pb(ii) removal efficiency.

### 3.3 Fixed bed breakthrough curve model

The adsorption behavior of hydrogel-II in a fixed bed for Pb(ii) is described using three models of Dose–response, Thomas, and Yoon–Nelson. The Dose–response model is an empirical model that describes the kinetics of adsorption in a fixed bed. To better utilize the adsorbent and sample solution, the maximum adsorption capacity of the adsorbent is also a crucial factor to consider in the design.^25^ The Thomas model is used for calculating the maximum adsorption capacity of a fixed bed and is one of the most commonly used models for studying breakthrough curves, assuming conformity to Langmuir isotherms and pseudo-second-order reaction kinetics.^24^ This model is applicable to adsorption processes where neither external nor internal diffusion limits the rate.^24^ The Yoon–Nelson model postulates that the likelihood of a reduction in the adsorption rate of the adsorbate on the adsorbent correlates proportionally with the probability of the adsorbate permeating through the fixed bed.^26^ This model is not only relatively straightforward mathematically, but it also lacks stringent requirements regarding the characteristics of the adsorbate, the type of adsorbent, or the physical properties of the fixed bed.^26^ Additionally, although the Yoon–Nelson and Thomas models have different model parameters, due to the same mathematical structure, the values of the parameters for these models obtained from fitting may overlap.

Upon examining the correlation coefficients (*R*^2^), a comparison revealed that the Thomas model (0.800–0.924) and the Yoon–Nelson model (0.800–0.924) exhibited more consistent values in comparison to the Dose–response model (0.564–0.947) as shown in Table 3. This suggests that the Thomas and Yoon–Nelson models provide a better fit for describing the Pb(ii) breakthrough curves than the Dose–response model. The adsorption amount (*q*_0_) in the Thomas model (Table 3) is close to the experimental adsorption amount (*q*) at *t*_s_ (Tables 1 and 2). Under different operating conditions (varying inflow rates, inflow directions, Pb(ii) solution concentrations, and fixed bed packing heights) (Table 3), the *k*_TH_ values of the Thomas model were almost identical, and closer to 0.006 L min^−1^ mg^−1^, indicating that Pb(ii) solution volume passing through a unit mass of hydrogel-II per unit time is similar. Furthermore, increasing the inflow rate, using a top inflow mode, increasing the initial concentration of Pb(ii), and decreasing the fixed bed packing height (Table 3) led to a downward trend in *τ* values, which might be due to the fixed bed reaching adsorption saturation more quickly, indicating insufficient contact between hydrogel-II and the Pb(ii) solution.

**Table 3:** Fitting parameters of the Dose–response, Thomas, and Yoon–Nelson models

Parameters	Dose–response model			Thomas model			Yoon–Nelson model		
Inflow rates	*a*	*q*_0_	*R* ^2^	*k*_TH_	*q*_0_	*R* ^2^	*k*_YN_	*τ*	*R* ^2^
0.159	1.04	35.9	0.739	0.0005	44.6	0.919	0.005	608	0.919
0.255	1.27	25.4	0.922	0.0006	42.5	0.896	0.006	346	0.896
0.318	1.38	24.9	0.947	0.0006	26.5	0.800	0.006	300	0.800

**Inflow direction**									
Bottom inflow	1.04	35.9	0.739	0.0005	44.6	0.919	0.005	608	0.919
Top inflow	1.48	20.2	0.868	0.0007	32.0	0.923	0.007	403	0.923

***C***_**0**_ **of Pb(** **ii** **) (mg L^−^** ^**1**^ **)**									
5	1.57	31.9	0.582	0.001	40.5	0.916	0.008	700	0.916
10	1.04	35.9	0.739	0.0005	44.6	0.919	0.005	608	0.919
20	1.71	65.6	0.647	0.0004	86.5	0.924	0.008	543	0.924

**Filling height**									
20	1.52	22.1	0.929	0.0006	43.4	0.812	0.006	300	0.812
40	1.04	35.9	0.739	0.0005	44.6	0.919	0.005	608	0.919
60	1.35	109.9	0.564	0.0007	46.9	0.855	0.007	885	0.855

### 3.4 Fixed bed adsorption–desorption cycle experiments

**Fig. 6:**
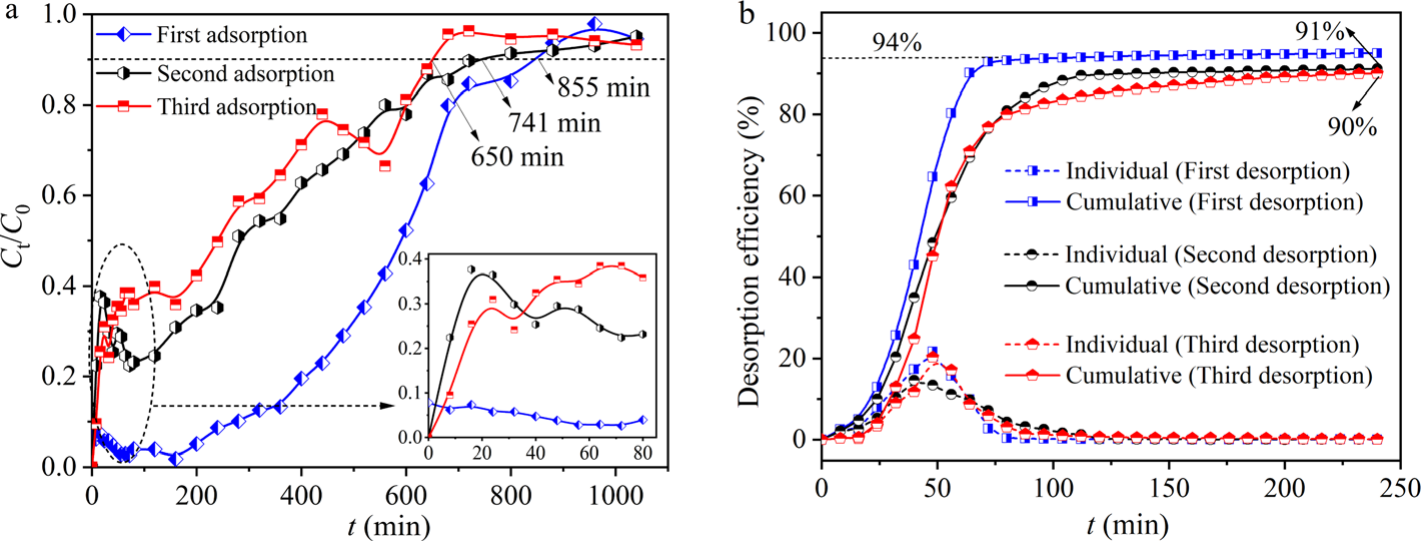
Regeneration ability of hydrogel-II in a fixed bed (a) adsorption and (b) desorption.

A 0.1 mol L^−1^ HCl solution was used as the eluent to regenerate hydrogel-II *in situ* in the fixed bed. Three consecutive adsorption–desorption cycle experiments were conducted. In the first adsorption, Fig. 6a shows that the breakthrough curve of hydrogel-II reached equilibrium after 855 min. In the first desorption experiment, Fig. 6b indicates that the cumulative desorption rate of Pb(ii) was 94%. In the second adsorption experiment, the breakthrough curve of hydrogel-II reached equilibrium after 741 min (Fig. 6a). Moreover, in the second desorption experiment, Fig. 6b exhibits that the cumulative desorption rate decreased slightly compared to the first desorption, at 91%. In the third adsorption experiment, Fig. 6a displays that the breakthrough curve reached equilibrium at 650 min. At the same time, the cumulative desorption rate was 90% in the third desorption experiment (Fig. 6b). The results indicate that hydrogel-II is relatively stable in the fixed bed and possesses certain regeneration and recycling capabilities, thereby demonstrating its potential application prospects in the treatment of Pb(ii) wastewater.

### 3.5 FT-IR analysis

**Fig. 7:**
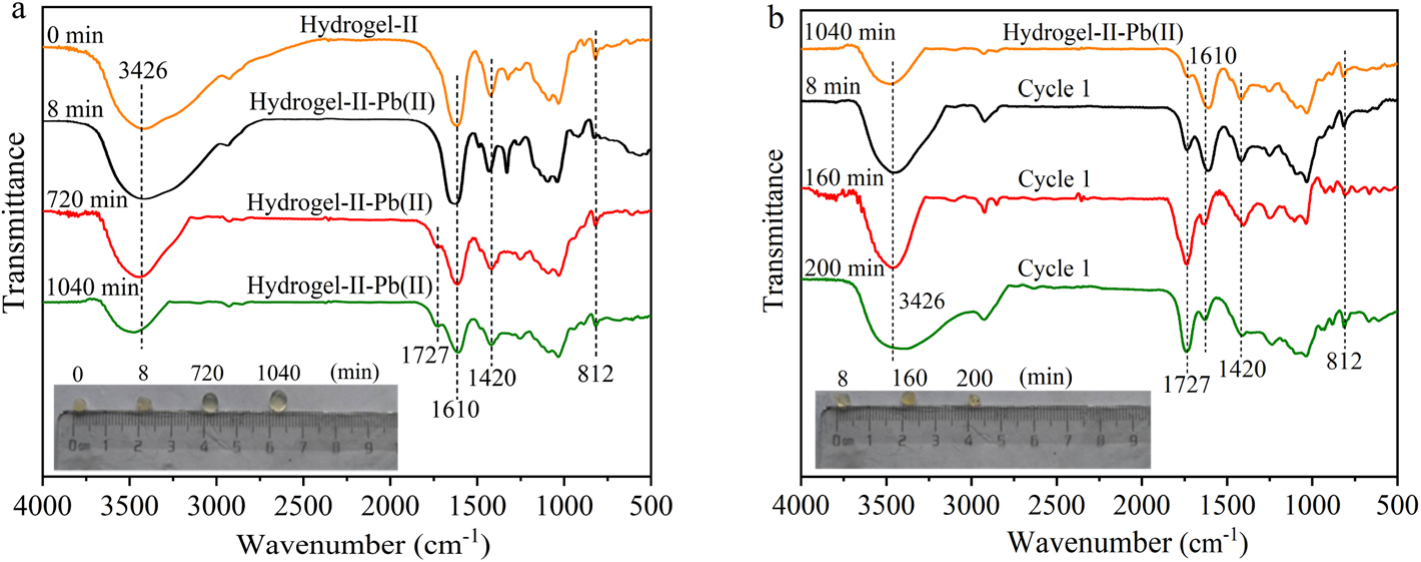
FT-IR spectrum (a) first adsorption and (b) first desorption (illustrated as images of particles at different times).

To further understand the removal of Pb(ii) by hydrogel-II in the fixed bed column, FT-IR spectroscopy was employed to study the functional group information during the adsorption–desorption cycle process. The absorption peaks of hydrogel-II at 3426, 1610, 1420, and 812 cm^−1^ correspond to the groups –OH, –COO–, C–H in the aromatic skeleton, and out-of-plane bending vibrations of symmetric triazine C_3_N_3_,^38^ respectively. As shown in Fig. 7a, during the first adsorption cycle, the four absorption peaks of hydrogel-II centered at 3426, 1610, 1420, and 812 cm^−1^ all weakened at 8, 720, and 1040 min, confirming that the functional groups –OH, –C=O, C–H in the aromatic skeleton, and out-of-plane bending vibrations of symmetric triazine C_3_N_3_ were involved in adsorption of Pb(ii). Additionally, in the first adsorption cycle, a new absorption peak at 1727 cm^−1^ appeared at 720 and 1040 min of adsorption time, attributing to protons (H^+^) existed in solution with a pH of around 6.0. Thus, these H^+^ ions can combine with the –COO– groups carried by hydrogel-II to form –COOH.^39^ At the same time, it shows that in the first desorption, the absorption peaks at 3426, 1610, 1420, and 812 cm^−1^ gradually increased as desorption time was prolonged (Fig. 7b). Moreover, the absorption peak at 1727 cm^−1^ also gradually intensified, consistent with the analysis during the first adsorption cycle, due to the presence of more H^+^ ions in solution during desorption process, leading to a more apparent increase in this peak.

**Fig. 8:**
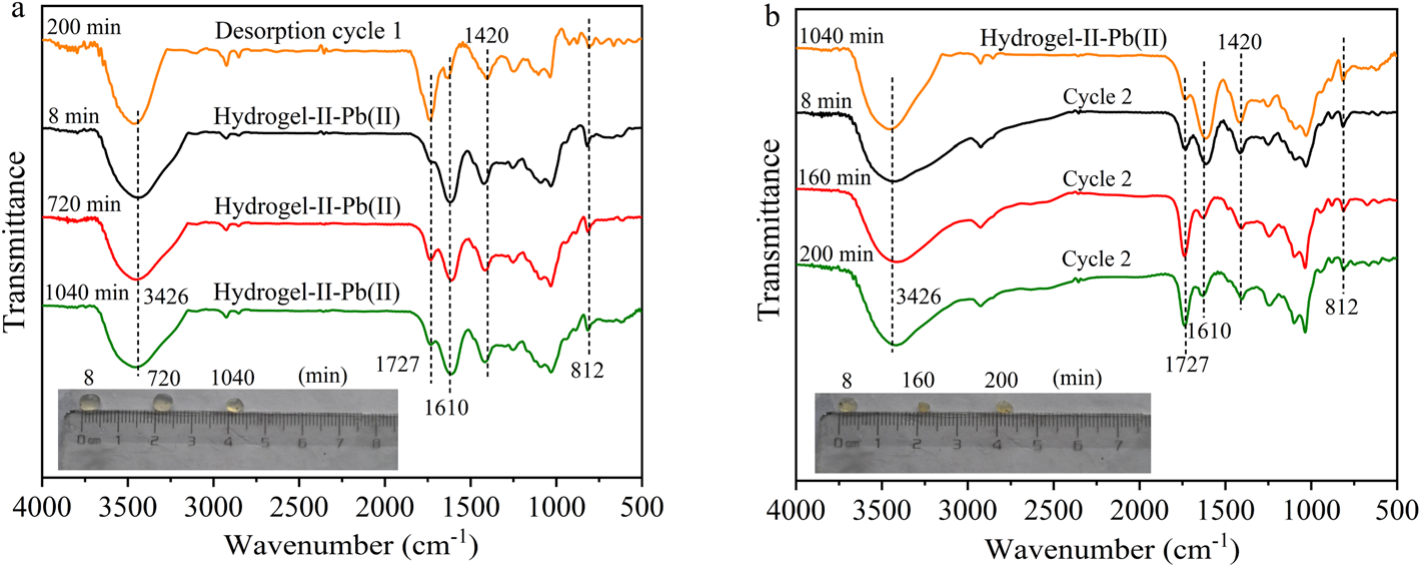
FT-IR spectrum (a) second adsorption and (b) second desorption (illustrated as images of particles at different times).

**Fig. 9:**
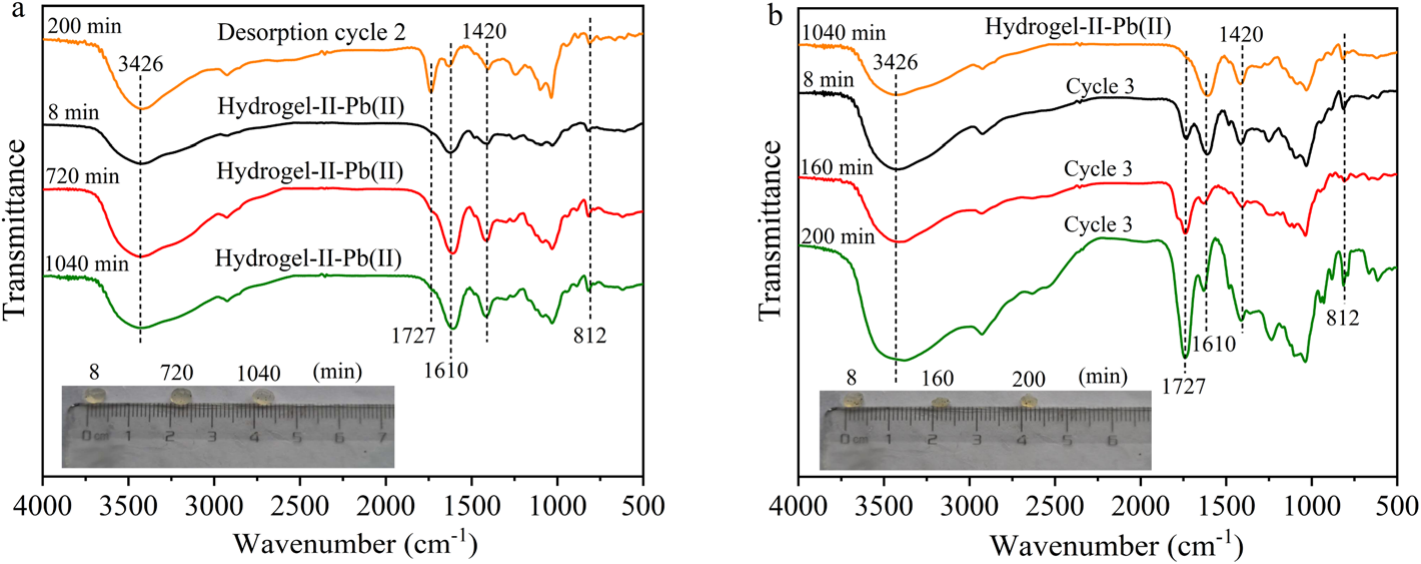
FT-IR spectrum (a) third adsorption and (b) third desorption (illustrated as images of particles at different times).

**Fig. 10:**
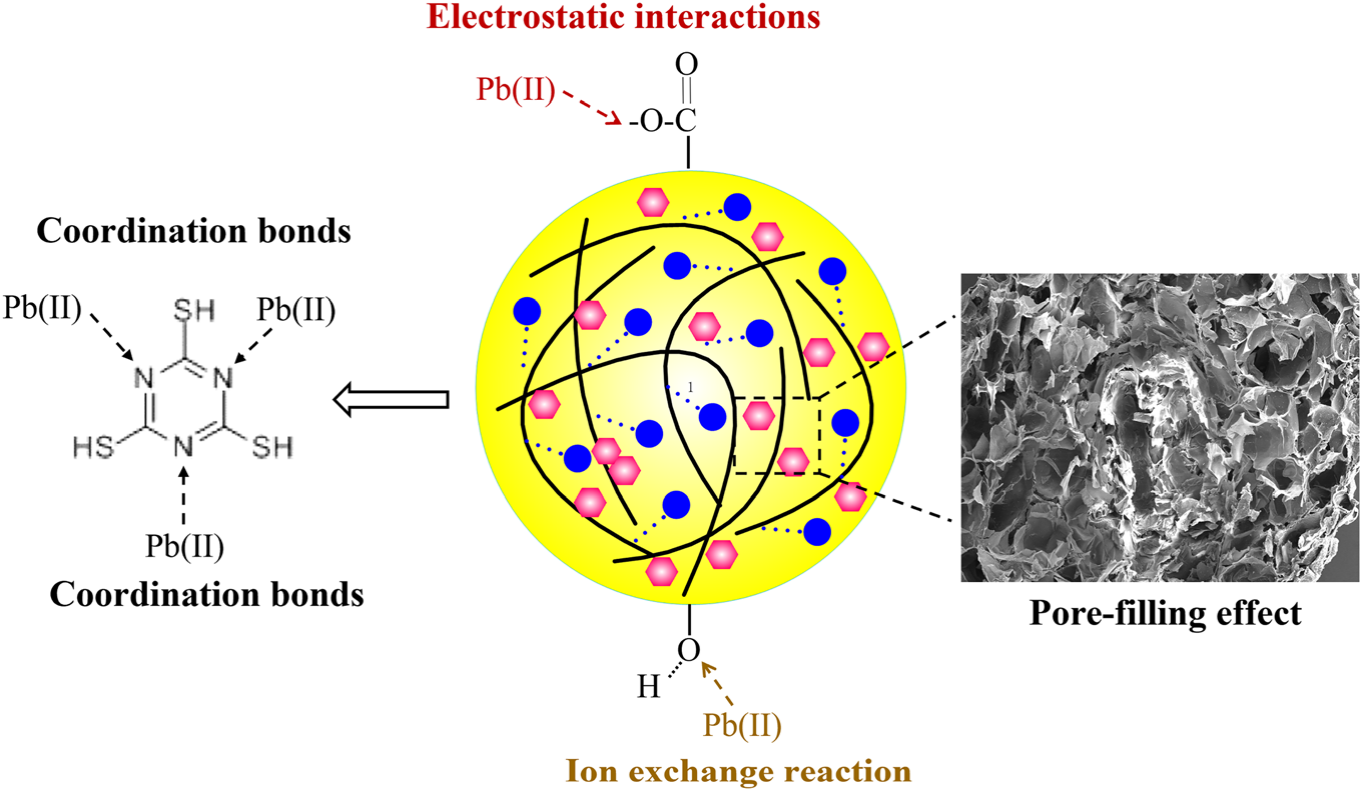
Mechanism diagram of hydrogel-II for Pb(ii) adsorption.

In the second and third adsorption–desorption cycles (Fig. 8 and 9), similar experimental phenomena were observed as in the first adsorption–desorption cycle. The experimental results suggest that the functional groups –OH, –COO, and the symmetric triazine ring in hydrogel-II may be the primary active sites for Pb(ii) adsorption. Based on the above analysis, the mechanism of Pb(ii) adsorption by hydrogel-II can be mainly summarized into the following four aspects (Fig. 10): (1) the –OH groups in hydrogel-II undergo ion exchange reactions with Pb(ii), thereby forming inner–sphere complexes;^40^ (2) the negatively charged –COO– functional groups in hydrogel-II engage in electrostatic interactions with Pb(ii);^41^ (3) the nitrogen atoms in the symmetric triazine rings of hydrogel-II, which possess lone pairs of electrons, can form coordination bonds with Pb(ii), thereby achieving Pb(ii) removal;^42^ and (4) according to SEM results (Fig. 3b), hydrogel-II has a porous structure. So a pore-filling effect may also exist in the process of Pb(ii) removal.^43^

By comparing the morphology of hydrogel-II beads during the adsorption–desorption cycles (illustrations in Fig. 7a, 8a, and 9a), compared to hydrogel-II beads before Pb(ii) adsorption, the beads were found to swell as adsorption time extended from 8 to 1040 min. However, in the desorption cycle experiments, the beads were slightly contracted compared to adsorption cycle (illustrations in Fig. 7b, 8b, and 9b). According to analysis, a possible reason for the above phenomena is that during adsorption process, hydrogel-II exhibits hydrophilicity, especially under the condition of an inflow solution pH of 6.0, triggering swelling phenomena. However, during the desorption process, using HCl as the desorbent made the solution acidic, and the abundance of H^+^ ions in solution causes carboxylation of the –COO– groups in hydrogel-II to form –COOH. Due to the reduced repulsion between protonated carboxyl groups, the solubility of alginate in water decreases, thereby enhancing the hydrophobicity of hydrogel-II. Therefore, during the desorption process, there was a phenomenon of hydrogel-II particle size reduction again.^44,45^ Through this mechanism, the mechanical strength of hydrogel-II can be improved, thereby prolonging its service life.^44,45^

## 4 Conclusion

This study explores the capacity of a fixed bed to remove Pb(ii) under various operating conditions and details the adsorption efficacy of hydrogel-II for Pb(ii), utilizing breakthrough curves for analysis. Through three consecutive adsorption–desorption cycle experiments, the regenerative ability of hydrogel-II was explored. The results show that: (1) regeneration experiments and fixed bed column experiments demonstrate that hydrogel-II can serve as an effective adsorbent for Pb(ii) removal from water; (2) compared to the Dose–response model, the Thomas model and Yoon–Nelson model both better describe the breakthrough curves; (3) when inflow rate of Pb(ii) is 0.159 L min^−1^, bottom inflow, the initial Pb(ii) concentration is 10 mg L^−1^, and hydrogel-II packed height is 40 cm, the adsorption capacity (*q*_0_) of hydrogel-II for Pb(ii) was determined to be 42.2 mg g^−1^; (4) increasing the packing height of the fixed bed, reducing the inflow rate of the fixed bed, lowering initial Pb(ii) concentration, and setting the inflow direction to bottom inflow, the maximum adsorption capacity of Pb(ii) overall shows an increasing trend, which is beneficial for Pb(ii) removal from water or wastewater; (5) the adsorption capacity of regenerated hydrogel-II still maintains good performance after three adsorption–desorption cycles, indicating that hydrogel-II has potential application prospects in the treatment of Pb(ii).

## Data availability

Data will be made available on request.

## Author contributions

Qiaorui Wang: writing – review & editing, conceptualization, investigation, methodology, software, formal analysis, data curation, funding acquisition. Dingyun Liang: formal analysis. Yalan Yang: formal analysis. Yunran Zhang: formal analysis. Yirong Wang: formal analysis. Lilong Zhang: methodology, software, formal analysis. Rui Ma: formal analysis. Zhirui Niu: writing – review & editing, conceptualization, methodology, validation, resources, supervision, project administration, funding acquisition.

## Conflicts of interest

There are no conflicts to declare.
